# Proper pertussis vaccination will probably not increase vaccination coverage: a case–control study

**DOI:** 10.1017/S0950268819001444

**Published:** 2019-08-20

**Authors:** R. Solano, A. V. Sanchez-Callejas, M. I. Alvarez-Ibañez, M. Sandiumenge-Durán, M. I. Fernández-San-Martín

**Affiliations:** 1 Unitat d'Avaluació, Sistemes d'Informació i Qualitat Assistencial, Gerència Territorial de Barcelona, Institut Català de la Salut, Barcelona, Spain; 2 Centre d'Atenció Primària La Sagrera, Gerència Territorial de Barcelona, Institut Català de la Salut, Barcelona, Spain; 3 Centre d'Atenció Primària Poble Nou, Gerència Territorial de Barcelona, Institut Català de la Salut, Barcelona, Spain

**Keywords:** *Bordetella pertussis*, epidemiology, preventable diseases, vaccine preventable diseases, vaccines

## Abstract

Vaccination coverage (VC) against pertussis can increase when management practices and policies at primary care centres (PCCs) are reinforced. From 2011 to 2015, we performed a case–control study to evaluate VC among pertussis patients treated at PCCs in Barcelona, Spain. We recorded pertussis in patients from 8- to 16-year-olds at 52 PCCs. Pertussis cases had laboratory diagnostic and controls were healthy outpatients visiting the same facility for reasons other than cough. DTaP/dTap VC was recorded as either proper vaccination status (five doses recorded) or improper vaccination status (<5 doses recorded). We used a logistic regression model to estimate OR and 95% CI. We included 229 cases and 576 controls. VC was higher in cases (mean 5.01, s.e.: 0.57) than in controls (4.89, s.e.: 0.73). Around 69% of the cases had received DTaP primary immunisation after 2–5 years and 31.4% of cases had the dTap booster immunisation after 7–10 years. The 87% of children 5–9 years were properly vaccinated. We found no protection from becoming ill among properly vaccinated children (OR 1.87; 95% CI 1.22–2.85). The highest VC was observed in patients with confirmed pertussis, which was likely due to a more exhaustive follow-up of the VC in these patients. Being properly vaccinated against pertussis will probably not increase VC.

## Background

Pertussis (whooping cough) is a disease with very high morbidity that is still persistent in countries with broad (>95%) vaccination coverage (VC), with epidemic periods (~3–5 years) in spite of the introduction of vaccination with a diphtheria-tetanus-pertussis-whole-cell (DTwP) or with a diphtheria-tetanus-acellular pertussis (DTaP) vaccines [1].

Pertussis mortality rate in children has increased from 195 000 worldwide in 2008, to 600 000 2018, with a higher incidence in unvaccinated children [2]. Pertussis affects all age groups, but is most common in children under 1 year of age [2, 3]. Despite the high primary VC (>90%) in some Central and Eastern European countries, the disease distribution has shifted towards other age groups, affecting between 9% and 40% of adolescents and adults, respectively [4–6].

In Spain, the current practice uses various acellular vaccines constituted with *Bordetella pertussis* antigens. These are combined trivalent, pentavalent or hexavalent vaccines bearing antigens that confer immunity to other diseases. Depending on the amount of antigen used, they can have high antigen load DTaP, which are used in primary vaccination series, or reduced-antigen-content tetanus-diphtheria-acellular (dTap), which provides a booster vaccination against pertussis [7]. Since 2002, Spain has been administering the acellular vaccine in a 2–4–6 month primary vaccination schedule, followed by two boost doses, the first at 18 months and the second between ages 4 and 6 years [8].

There is evidence that management strategies and protocols applied in the health centres can have a direct effect on VC [9]. Sending regular reminders in the form of mobile phone text messaging to patients (or to patients’ tutors) to get vaccinated achieved a 5–20% increase in VC [9]. In addition, improvements in immunisation registries in the health care centres have also been associated with higher VC in adolescents [10, 11]. For example, immunisation registries in Spain allow researchers to use individual data to conduct studies of vaccine effectiveness and to efficiently monitor VC.

According to the recommendations from the World Health Organization (WHO), there should be >90% coverage with primary series and booster vaccinations with DTaP/dTap. However, the data on VC from Spain showed that reinforcement coverage reached only 82% in 2016 [8].

Since the distribution of pertussis among younger people has progressed towards older age groups, it has now become necessary to evaluate and improve current vaccination strategies and the available acellular vaccines [12]. The objective of our study was to evaluate VC in patients aged 8–16 years who had been diagnosed with pertussis, and to compare these results to VC in healthy control subjects.

## Methods

### Study design

We designed a case–control study of 8- to 16-year-old patients with confirmed pertussis who had attended one of 52 Barcelona primary care centres (PCCs) in the Catalan Health Institute (ICS) between 2011 and 2015. Vaccination status was verified from the cases' and controls' digital clinical records e-CAP/MEAP (these acronyms refer to the digital system used in Catalan PCCs to record patients' medical data).

### Case definition

All confirmed diagnoses of pertussis among 8- to 16-year-olds during the study period that were recorded in the e-CAP/MEAP (ICD10 codes: A37.0, A37.1, A37.8, A37.9). A case was considered confirmed if infection with *B. pertussis* was positive by laboratory diagnostic tests. The laboratory diagnostic methods used in Catalonia are: the identification of *B. pertussis* in culture (Bordet–Gengou or Regan–Lowe) and a reactive polymerase chain reaction (PCR) [13, 14]. Pertussis biological detection was carried out by many clinical laboratories in Spain and in other European countries. These tests generally used the IS481 sequence and its isoform IS1002, since they are specific for *B. pertussis* and have multiple copies, which increase the sensitivity of the test [15, 16]. According to the National Epidemiological Surveillance Network (RENAVE in Spanish), a confirmed case of pertussis is defined as a laboratory-confirmed disease (microbiological isolation or genome detection by amplification techniques of *B. pertussis* in nasopharyngeal swabs), plus the presence of clinically compatible disease symptoms (cough ⩾2 weeks, and at least one of the following symptoms: paroxysmal coughing, inspiratory stridor, vomit after coughing, ⩾37 °C body temperature or apnoea) [13].

### Control definition

For each case, we selected three controls from among the healthy patients visiting the same PCCs during the same study period, according to the e-CAP/MEAP registry. Controls had the same age distribution as cases (±6 months) and sex was balanced between the two groups. Only individuals who had attended the PCCs for reasons other than cough were included in the study. Given that pertussis is not associated with any underlying diseases, this variable was not collected for either of the two groups, and was not analysed.

### Exclusion criteria

We excluded patients who had previously shown symptoms compatible with pertussis (e.g. cough ⩾2 weeks, paroxysmal cough or vomit after coughing) and subjects from private PCCs. Cases and controls who had previously been diagnosed with pertussis in e-CAP/MEAP were excluded from our study.

### Variables collected from the e-CAP/MEAP

Demographic: sex and age were divided into two groups: 8–12 years and 13–16 years resulting in 50% of the patients in each group.

Clinical: date of onset of symptoms, date of diagnosis, clinical manifestations registered by professionals in the e-CAP/MEAP.

Complications: pneumonia, seizures, encephalopathy, bronchitis, tachypnoea, respiratory syncytial virus, hospital admission. Before assigning people to the study group or control group, the e-CAP/MEAP was verified to check that the patient had been given the differential diagnosis of bronchitis and viral respiratory infections. Laboratory diagnostic tests included culture tests and/or positive/negative PCR tests.

DTaP/dTap VC status was recorded as either proper vaccination status (i.e. individuals fully immunised with five doses recorded) or improper vaccination status (i.e. individuals partially immunised with <5 doses recorded).

### Statistical methods

We conducted a descriptive comparative analysis of the socio-demographic characteristics and the number of doses of pertussis vaccines administered to cases and controls, using percentages, mean scores and measures of statistical dispersion. In cases with small samples sizes, we used the Fisher's exact test.

We used a logistic regression model to estimate the odds ratios (OR) and 95% confidence intervals (95% CI) for the confirmed pertussis cases and the controls, based on the number of doses of pertussis vaccine administered. All statistical analyses were performed using the Statistical Package for Social Sciences (SPSS^®^ version 18.0, Chicago, IL, USA) for Windows (Microsoft Corp., Redmond, WA, USA). Assuming a case:control ratio of 1:3, we compute the sample size, using GRANMO software (Version 7.12, IMIM, Barcelona, Spain) [17].

### Ethical considerations

e-CAP/MEAP data for the selected patients were entered into a new, password-protected database. Subjects were later identified using an anonymous encrypted code, which was maintained throughout the study. The researchers guaranteed the confidentiality of the results. The study has been approved by the National Clinical Research Ethics Committee *Jordi Gol i Gurina*.

## Results

A total of 52 PCCs of ICS attended 87 938 patients of 8–16 years old from 2011 to 2015. We included 229 cases and 576 controls. A total of 52.4% of the cases were female. In our study, 68.6% of the cases had received DTaP primary immunisation after 2–5 years, and of these, 31.4% had received the dTap booster immunisation after 7–10 years. The following clinical symptoms compatible with pertussis were observed in the cases: coughing for ⩾2 weeks (38.9%), vomiting after coughing (22.7%), paroxysmal coughing (19.2%), body temperature >37 °C (15.3%), inspiratory stridor (11.8%) and cyanosis (4.4%). The incidence of inspiratory stridor, a symptom associated with pertussis, was significantly higher in the study group than in controls (*P* < 0.001). In addition, people in the case group were more likely to suffer clinical symptoms compatible with pertussis than people in the control group (Table 1).

**Table 1.** Analysis of demographic and clinical variables associated with pertussis in cases and control subjects

	Cases		Controls		*P* value
	*n* (229)	%	*n* (576)	%	
Sex					
Male	109	47.6	288	50.0	
Female	120	52.4	288	50.0	
Age group					
8–12 years	157	68.6	379	65.8	
13–16 years	72	31.4	197	34.2	
Registered vaccine doses. Average (s.e.)	5.01 (0.57)		4.89 (0.73)		0.019
Compatible with pertussis symptoms					
Cough ⩾2 weeks	89	38.9	–		
Vomit after coughing	52	22.7	–		
Paroxysmal coughing	44	19.2	–		
Cyanosis	10	4.4	–		
Inspiratory stridor	27	11.8	2	0.3	<0.001
Body temperature >37°	35	15.3	82	14.2	0.74
Concomitant diseases					
Pneumonia	7	3.3	16	2.2	0.680
Seizures	0		0		
Bronchitis	50	21.8	84	14.6	0.016
Co-infection	28	12.2	2	0.3	<0.001

s.e., standard error.

During the study period, 21.8% of cases presented with bronchitis, 12.2% presented with co-infection and 3.3% presented with pneumonia. Bronchitis was more common among cases than controls (21.8% *vs.* 14.6%, *P* = 0.016). A negative diagnosis of pertussis was confirmed for people in the control group who had been diagnosed with bronchitis in the e-CAP/MEAP.

Twelve per cent of cases had co-infection diagnosed by laboratory tests. While only 20.0% of cases had a positive culture test for pertussis, 98.4% had shown positive genome detection of *B. pertussis* in the nasopharyngeal smear. Twenty-eight per cent of cases were referred to the hospital to be seen by another specialist. DTaP/dTap VC was higher in cases (mean 5.01, s.e.: 0.57) than in controls (4.89, s.e.: 0.73) (*P* = 0.02). VC among 14-year-old children in Catalonia was 96.4%. Eighty-seven per cent of children aged 5–9 years received complete vaccination with five doses of the DTaP/Tdap vaccines.

Ninety per cent of cases had a proper vaccination status compared to 81.3% of controls (OR 1.87, 95% CI 1.22–2.85; *P* = 0.03; Table 2). According to e-CAP/MEAP data, patients were vaccinated at the appropriate time points according to the corresponding vaccination schedule, as well at the appropriate time points according to age. The time at which a vaccine was administered to a patient was recorded. Among women, cases were more likely than controls to have proper vaccination status (OR 2.60, 95% CI 1.33–5.07; Table 2); in contrast, these differences in VC were not statistically significant between male cases and controls (OR 1.41, 95% CI 0.81–2.43).

**Table 2.** Vaccination coverage according to sex and age group in cases and control subjects, Barcelona PCCs, Spain

	Vaccination status						
	Proper		Improper		*P* value	OR	CI 95% OR
	*N*	%	*N*	%			
Cases	206	90.0	23	10.0	**0.03**	**1.87**	**1.22–2.85**
Controls^a^	468	81.3	108	18.8			
Male							
Cases	95	87.2	14	12.8	0.21	1.41	0.81–2.43
Controls^a^	236	81.9	52	18.1			
Female							
Cases	111	92.5	9	7.5	**0.03**	**2.60**	**1.33–5.07**
Controls^a^	232	80.6	53	19.4			
8–12 years							
Cases	142	90.4	15	9.6	**0.02**	**2.15**	**1.28–3.62**
Controls^a^	301	79.4	578	20.6			
13–16 years							
Cases	64	88.9	8	11.1	0.39	1.37	0.66–2.85
Controls^a^	167	84.8	30	15.2			
Recorded vaccine doses. Average (s.e.)	5.01 (0.57)		4.89 (0.73)		0.019		

s.e., standard error. Bold indicates the variable is statistically significant *p* < 0.05.

a Referent group.

In terms of age, we observed a significant association between vaccination status and pertussis among 8- to 12-year-olds (*P* = 0.02; OR 2.15, 95% CI 1.28–3.62) but not among 13- to 16-year-olds (*P* = 0.39; OR 1.37, 95% CI 0.66–2.85), indicating that younger controls are more likely to have been properly vaccinated than older controls (Table 2).

## Discussion

Since its introduction in 2012, the boost vaccines (dTap/dT) have had low VC nationally, resulting in a high percentage of pertussis cases among ⩾15-year-olds [18]. This finding is consistent with the results of our study; where the VC among cases was lower in 13- to 16-year-olds (88.9%) than among 8- to 12-year-olds (90.4%). In all clinical trials, DTaP/dTap vaccines were described as slightly less effective and as being protective for a shorter time period (<2 years after the third injection) [19, 20].

In our study, VC was higher in the cases than in the controls, likely due to the more careful monitoring of vaccination status among children with confirmed pertussis, and higher VC in this group. There is evidence that immunity against pertussis after vaccination is quite high for the first year of life, when anti-pertussis vaccines are administered for primary immunisation in infancy at 2, 4 and 6 months of age, which was the vaccination schedule followed by individuals in our case and control groups. Similarly, another study showed that DTaP vaccines have 84% protective efficacy in the first 2 years of life [21].

It is now well known that the immunity induced by the DTaP vaccine lasts around 5–10 years according to the epidemiological situation report (WHO position paper). This is supported by a recent report on the data paediatric ambulatory surveillance of confirmed pertussis in France [22], and a recent publication based on 16 years of disease-specific surveillance in Massachusetts [23]. It is well known that immunity from pertussis after vaccination reaches high levels during the first year, and then declines thereafter [24, 25]. While in this study we did not analyse the time elapsed since vaccination and the duration of immunity provided by the vaccine, the data for the cases indicated that the time elapsed for the primary immunisation and booster vaccination was 2–5 and 7–10 years, respectively.

Our findings suggest that vaccination does not protect from developing pertussis disease. This is consistent with the findings of another study showing that vaccination with a fifth dose of dTap appeared to have a shorter protection period and did not reduce the risk of becoming ill or getting infected [25].

VC as one indicator of the effectiveness of vaccination programmes should be thoroughly evaluated in pertussis cases. When vaccination records are not available, a periodic evaluation report should be carried out to monitor the shortcomings in VC and provide corrective actions [26].

In this study, we observed a high proportion of correct vaccination among pertussis cases (90.0%), compared to a study conducted in Minnesota which showed a lower VC with dTap (58.2%) [27]. Nonetheless, these studies are essential for identifying other relevant socio-demographic variables, which we did not include in our analysis (e.g. characteristics of the population attending the PCCs, urbanity, socio-economic deprivation index, educational level and parents' country of origin), which can influence the coverage and effectiveness of vaccines, and can provide valuable input for developing more successful vaccination strategies [28].

The most common symptoms consistent with pertussis were cough for ⩾2 weeks (38.9%), vomiting after coughing (22.7%) and paroxysmal coughing (19.2%). These results are similar to those observed in a US case–control study conducted in 1998–2014 to evaluate the risk factors associated with infant death from pertussis [29]. A recent study showed that while pertussis acellular vaccines (DTaP or dTap) prevent disease symptoms, they do not prevent *B. pertussis* infection/colonisation and the subsequent chain of disease transmission [30].

Although immunity declines over time, immunised people who develop pertussis later on tend to suffer from less severe symptoms. As highlighted by another study, serious pertussis symptoms and complications were less common among pertussis patients who had received an age-appropriate number of pertussis vaccines, thus demonstrating that the positive impact of pertussis vaccination extends beyond decreasing the risk of disease [31].

Bronchitis was the most common disease complication in our study (21.8%). *B. pertussis* infection can cause life-threatening complications and exacerbate concomitant chronic diseases, especially in vulnerable groups such as children and adolescents who have not received booster vaccines, patients with immunodeficiency or pulmonary complications, and health care professionals exposed to contagious diseases [32]. Norway and Spain have reported a high anti-pertussis VC; disease complications have been widely reported, including co-infection with respiratory pathogens other than *B. pertussis* [33, 34].

In this sense, protection from pertussis infections following booster vaccination with dTap has a limited duration, regardless of the type of anti-pertussis vaccines received during childhood [35, 36]. In our study, VC among cases with confirmed pertussis was higher than among healthy controls, showing that proper pertussis vaccination will probably not increase VC. This could be interpreted as if humoral and cell-mediated immunity persisted, regardless of how antibodies deteriorate. As other authors have stated, the DTaP vaccine may enhance the cell-mediated immune response, although additional studies are needed to verify this [37, 38].

We found that VC was limited in 8- to 16-year-old patients, highlighting the need for effective vaccination programmes in this age group. We recommend that this need be met through preventive, health promotion and educational programmes at the PCCs [22, 39].

Several limitations to the present study should be noted. First, the information bias and registration errors in e-CAP/MEAP. Second, laboratory tests for confirming pertussis cases may have been carried out in a sporadic manner at the PCCs, such that some cases with an atypical clinical symptomatology could have remained undetected, which complicates the estimations of the precise incidence of pertussis. However, we checked that the controls did not have a previous pertussis diagnosis to minimise selection bias. The e-CAP/MEAP records were carefully analysed for specific case definitions, to ensure that erroneous or false-positive cases were not included in the analysis.

In conclusion, we observed an overall VC of >81% among patients with pertussis and healthy controls aged 8–16 years, and even higher among the cases. Despite the fact that PCCs recommend booster vaccination for 8- to 16-year-old population, the VC for primary and secondary anti-pertussis boosters was <90%. This could be attributed to the presence of one or more potential unobserved confounders. We conclude that the findings in this study are unusual and require further studies to find a plausible explanation. Performing the same study in different countries could help to explain our observations.

## References

[1] Chambers C (2014) Pertussis surveillance trends in British Columbia, Canada, over a 20-year Period: 1993–2013. Canada Communicable Disease Report 40, 31–41. 10.14745/ccdr.v40i03a02 PMC5864486 29769880

[2] Black RE (2010) Pertussis vaccines: WHO position paper. The Weekly Epidemiological Record 85, 385–400.20939150

[3] Paksu MS (2013) Fulminant pertussis in very young infants: two cases and review of the literature. The Turkish Journal of Pediatrics 55, 426–429. 24292037

[4] Díez-Domingo J (2004) Incidence of pertussis in persons < or =15 years of age in Valencia, Spain: seroprevalence of antibodies to pertussis toxin (PT) in children, adolescents and adults. Journal of Infection 49, 242–247. 15337342 10.1016/j.jinf.2004.03.003

[5] Gil A (2001) Hospital admissions for pertussis in Spain, 1995–1998. Vaccine 19, 4791–4794. 11535331 10.1016/s0264-410x(01)00213-4

[6] Comité asesor de vacunas de la Asociación Española de Pediatría (2006) Calendario de vacunación de la Asociación Española de Pediatría: recomendaciones 2006. Anales de Pediatría 64, 74–77. 16539920 10.1016/s1695-4033(06)70012-1

[7] Grupo de trabajo tos ferina 2012 (2013) De la ponencia de programas y registro de vacunaciones. Revisión del programa de vacunación frente a tos ferina en España. Comisión de Salud Pública del Consejo Interterritorial del Sistema Nacional de Salud. Ministerio de Sanidad, Servicios Sociales e Igualdad. Available at https://www.msssi.gob.es/profesionales/saludPublica/prevPromocion/vacunaciones/difteria_tetano_tosferina.htm (Accessed 11 March 2018).

[8] Ministerio de Sanidad, Servicios Sociales e Igualdad. Coberturas de vacunación. Datos estadísticos. Available at https://www.msssi.gob.es/profesionales/saludPublica/prevPromocion/vacunaciones/coberturas.htm (Accessed 20 July 2018).

[9] Briss PA (2000) Reviews of evidence regarding interventions to improve vaccination coverage in children, adolescents, and adults. The Task Force on Community Preventive Services. American Journal of Preventive Medicine 18, 97–140. 10.1016/s0749-3797(99)00118-x 10806982

[10] Szilagyi PG (2000) Effect of patient reminder/recall interventions on immunization rates: a review. Journal of the American Medical Association 284, 1820–1827. 11025835 10.1001/jama.284.14.1820

[11] Fiks AG (2013) Effectiveness of decision support for families, clinicians, or both on HPV vaccine receipt. Pediatrics 131, 1114–1124. 23650297 10.1542/peds.2012-3122 PMC3666111

[12] World Health Organization. Immunization, vaccines and biologicals. Available at http://www.who.int/immunization/topics/pertussis/en/ (Accessed 12 July 2016).

[13] Centro Nacional de Epidemiología (2013) Instituto de Salud Carlos III. Red Nacional de Vigilancia Epidemiológica. Protocolos de enfermedades de declaración obligatoria. Madrid. Available at http://www.isciii.es/ISCIII/es/contenidos/fd-servicios-cientifico-tecnicos/fd-vigilancias-alertas/fd-procedimientos/protocolos.shtml (Accessed 11 March 2018).

[14] Guiso N (2016) Surveillance of pertussis: methods and implementation. Expert Review of Anti-infective Therapy 14, 657–667. 27224518 10.1080/14787210.2016.1190272

[15] Espy MJ (2006) Real-time PCR in clinical microbiology: applications for routine laboratory testing. Clinical Microbiology Reviews 19, 165–256. 16418529 10.1128/CMR.19.1.165-256.2006 PMC1360278

[16] Guiso N (2011) What to do and what not to do in serological diagnosis of pertussis: recommendations from EU reference laboratories. European Journal of Clinical Microbiology 30, 307–312. 10.1007/s10096-010-1104-y PMC3034915 21069406

[17] Estimation of the sample size, software GRANMO version 7.12. Available at https://www.imim.cat/ofertadeserveis/software-public/granmo/ (Accessed 13 April 2013).

[18] Campins M (2013) Whooping cough in Spain. Current epidemiology, prevention and control strategies. Recommendations by the Pertussis Working Group. Enfermedades Infecciosas y Microbiología Clínica 31, 240–253. 23411362 10.1016/j.eimc.2012.12.011

[19] Taranger J (1997) Unchanged efficacy of a pertussis toxoid vaccine throughout the two years after the third vaccination of infants. The Pediatric Infectious Disease Journal 16, 180–184. 9041597 10.1097/00006454-199702000-00003

[20] Simondon F (1997) A randomized double-blind trial comparing a two-component acellular to a whole-cell pertussis vaccine in Senegal. Vaccine 15, 1606–1612. 9364690 10.1016/s0264-410x(97)00100-x

[21] Salmaso S (2001) Sustained efficacy during the first 6 years of life of 3-component acellular pertussis vaccines administered in infancy: the Italian experience. Pediatrics 108, E81. 11694665 10.1542/peds.108.5.e81

[22] Guiso N (2017) Whooping cough surveillance in France in pediatric private practice in 2006–2015. Vaccine 35, 6083–6088. 28974408 10.1016/j.vaccine.2017.09.072

[23] Domenech de Cellès M (2018) The impact of past vaccination coverage and immunity on pertussis resurgence. Science Translational Medicine 10, 434. 10.1126/scitranslmed.aaj1748 PMC6063734 29593103

[24] Radke S (2017) Age-specific effectiveness following each dose of acellular pertussis vaccine among infants and children in New Zealand. Vaccine 35, 177–183. 27866766 10.1016/j.vaccine.2016.11.004

[25] Rigo-Medrano MV (2016) Acellular vaccines (DTPa/dTpa) against whooping cough, protection duration. Enfermedades Infecciosas y Microbiología Clínica 34, 23–28. 10.1016/j.eimc.2015.01.014 25735715

[26] Robert E (2014) Vaccination coverage for infants: cross-sectional studies in two regions of Belgium. BioMed Research International 2014, 838907. 24971352 10.1155/2014/838907 PMC4058188

[27] Barber A (2017) Coverage with tetanus, diphtheria, and acellular pertussis vaccine and influenza vaccine among pregnant women – Minnesota, March 2013-December 2014. Morbidity and Mortality Weekly Report 66, 56–59. 28103212 10.15585/mmwr.mm6602a4 PMC5657652

[28] Hill HA (2016) Vaccination coverage among children aged 19–35 months – United States, 2015. Morbidity and Mortality Weekly Report 65, 1065–1071. 27711036 10.15585/mmwr.mm6539a4

[29] Winter K (2015) Risk factors associated with infant deaths from pertussis: a case-control study. Clinical Infectious Diseases 61, 1099–1106. 26082502 10.1093/cid/civ472

[30] Warfel JM (2014) Acellular pertussis vaccines protect against disease but fail to prevent infection and transmission in a nonhuman primate model. Proceedings of the National Academy of Sciences of the USA 111, 787–792. 24277828 10.1073/pnas.1314688110 PMC3896208

[31] McNamara LA (2017) Reduced severity of pertussis in persons with age-appropriate pertussis vaccination-United States, 2010–2012. Clinical Infectious Diseases 65, 811–818. 29017283 10.1093/cid/cix421 PMC5755965

[32] Zycinska K (2017) Whooping cough in adults: a series of severe cases. Advances in Experimental Medicine and Biology 955, 47–50. 28039663 10.1007/5584_2016_167

[33] Moreno Samos M (2015) Incidence and severity of pertussis in infants with a respiratory syncytial virus infection. Enfermedades Infecciosas y Microbioliología Clínica 33, 476–479. 10.1016/j.eimc.2014.09.009 25459193

[34] Reinton N (2013) Respiratory tract infections during the 2011 *Mycoplasma pneumoniae* epidemic. European Journal of Clinical Microbiology & Infectious Diseases 32, 835–840. 23354674 10.1007/s10096-013-1818-8

[35] Koepke R (2014) Estimating the effectiveness of tetanus-diphtheria-acellular pertussis vaccine (Tdap) for preventing pertussis: evidence of rapidly waning immunity and difference in effectiveness by Tdap brand. The Journal of Infectious Diseases 210, 942–953. 24903664 10.1093/infdis/jiu322

[36] Klein NP (2017) Waning protection following 5 doses of a 3-component diphtheria, tetanus, and acellular pertussis vaccine. Vaccine 35, 3395–3400. 28506516 10.1016/j.vaccine.2017.05.008

[37] Vermeulen F (2013) Persistence at one year of age of antigen-induced cellular immune responses in preterm infants vaccinated against whooping cough: comparison of three different vaccines and effect of a booster dose. Vaccine 31, 1981–1986. 23429006 10.1016/j.vaccine.2013.02.004

[38] Edelman KJ (2004) Pertussis-specific cell-mediated and humoral immunity in adolescents 3 years after booster immunization with acellular pertussis vaccine. Clinical Infectious Diseases 39, 179–185. 15307026 10.1086/421943

[39] Lu PJ (2017) Impact of provider recommendation on Tdap vaccination of adolescents aged 13–17 years. American Journal of Preventive Medicine 53, 373–384. 28495221 10.1016/j.amepre.2017.03.022 PMC5794009

